# Synthesis and structural characterization of the heavy tricysteinylpnictines, models of protein-bound As(iii), Sb(iii), and Bi(iii)†

**DOI:** 10.1039/d4dt02476a

**Published:** 2024-11-26

**Authors:** Sophia E. Hollow, Timothy C. Johnstone

**Affiliations:** a Department of Chemistry and Biochemistry, University of California Santa Cruz Santa Cruz California 95064 USA johnstone@ucsc.edu

## Abstract

The heavier group 15 elements As, Sb, and Bi are more restricted in their biochemistry than the nearly ubiquitous lighter congeners N and P, but organisms do encounter compounds of these elements as environmental toxins, starting materials for secondary metabolite biosynthesis, substrates for primary metabolism, or exogenously applied medicines. Under many physiological conditions, these compounds are transformed into pnictogen(iii) species, the soft Lewis acidic character of which leads them to interact strongly with biologically relevant soft Lewis bases such as small-molecule thiols or cysteine residues of proteins and peptides. The archetypal complexes As(Cys)_3_, Sb(Cys)_3_, and Bi(Cys)_3_ have been studied in the past but a lack of detailed information about their molecular structures has hampered the analysis of protein structures featuring As(iii), Sb(iii), and Bi(iii) bound to cysteine thiolate residues. In many cases, the formation of such protein adducts is proposed to play a key role in the mechanism of action of inorganic drugs that feature these elements. Here, we refine synthetic strategies to access As(Cys)_3_, Sb(Cys)_3_, and Bi(Cys)_3_, describe their crystal structures, analyze structural trends across the series and across Pn(SR)_3_ compounds deposited in the Cambridge Structural Database, and compare their features to the structures of proteins with these centers bound to Cys_3_ motifs. Significant differences were noted for many of the protein structures.

## Abstract 2

We synthesize and characterize As(Cys)_3_, Sb(Cys)_3_, and Bi(Cys)_3_, describe their crystal structures, analyze structural trends across Pn(SR)_3_ compounds, and compare their features to the structures of proteins with these centers bound to Cys_3_ motifs.

## Graphical abstract

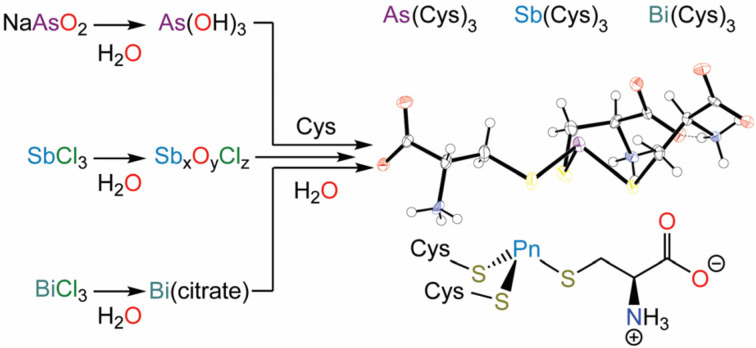

## Introduction

A sharp divide exists in the bioinorganic chemistry of the group 15 elements. N and P are ubiquitous and integral to all life as we know it, whereas the heaviest stable elements, Sb and Bi, have no known productive biochemistry. Compounds of these heavy elements can act as environmental toxins, however, and many organisms have developed detoxification strategies as a response. The dividing line between these spaces is occupied by As. It occurs readily in the environment, and it is toxic to many organisms, which has resulted in the evolution of As detoxification mechanisms. In some cases, the product of As detoxification can itself be a molecule that is toxic to other organisms and it has been proposed that some species have evolved to exploit the production of such toxins for evolutionary advantage.^1^ Indeed, some organisms have incorporated As into more elaborate secondary metabolites that may serve defense, predation, or detoxification purposes.^2–5^ As-based oxyacids/oxyanions can also serve as substrates for microbial respiration.^6,7^ In addition to these naturally occurring intersections between biology and the heavier group 15 elements, compounds of these elements are also introduced into biological systems as therapeutics.^8^ An As-based drug (arsphenamine) was the product of what could arguably be described as the first modern medicinal chemistry campaign, in which the structure of a molecule was systematically and rationally altered to improve efficacy.^9^ Currently, As-based drugs (*viz*., melarsoprol) are used to treat trypanosomiasis and arsenic trioxide (ATO) is used as a part of a frontline combination therapy for acute promyelocytic leukemia (APL). Sb-based drugs (*viz*., sodium stibogluconate and meglumine antimoniate) are used to treat leishmaniasis. Bi-based drugs (*viz*., bismuth subsalicylate and ranitidine bismuth citrate) are used to treat gastrointestinal discomfort. Medicinal inorganic chemistry research continues into new and extended applications of compounds of these elements.^8^

The biological effects of these compounds arise from their interactions with the small-molecule and macromolecular components of the cell. The reducing environment of the cytoplasm often results in these compounds being reduced to the +3 oxidation state. By definition, the Pn(iii) centers are softer Lewis acids than their Pn(v) counterparts. The absolute hardness values of atomic As, Sb, and Bi gradually decrease down the family,^10^ and all readily form complexes with thiolate ligands. Indeed, the mechanisms of action of As-, Sb-, and Bi-based therapeutics uniformly invoke the interaction of the Pn(iii) species with low-molecular weight thiols (*e.g.*, cysteine, glutathione, trypanothione) or cysteine residues of proteins.^11–13^

In most types of human cells, the small-molecule intracellular thiol of greatest abundance is glutathione (GSH). Consequently, many detailed spectroscopic and spectrometric studies of the interaction of GSH with As(iii), Sb(iii), and Bi(iii) have been performed and confirm the formation of Pn(SG)_3_ species with the ligand binding through the S atom of the cysteinyl residue.^14–18^ Interaction of these Pn(iii) species with proteins is typically proposed to occur predominantly through cysteine residues. Cysteine triads were identified as important motifs in the binding of As(iii) and Sb(iii) by the *ars*-encoded metalloid efflux machinery,^19^ the binding of Sb(iii) by the essential leishmanial enzyme trypanothione reductase,^20^ the anti-APL activity of ATO *via* interaction with the mutant fusion protein PML-RARα,^21^ and very recently in the binding of Bi(iii) to the SARS-CoV-2 helicase.^22^ Moreover, As(iii), Sb(iii), and Bi(iii) have all been confirmed to bind to cysteine-rich metallothionein.^23–26^ To better understand the structures of these complexes, model peptides that assembled into well-defined motifs capable of binding metal ions have been previously investigated. The structure of an As(iii)–peptide_3_ complex was investigated using X-ray crystallography and EXAFS, and a Bi(iii)–peptide_3_ complex was studied by EXAFS.^27–30^

**Scheme 1:**
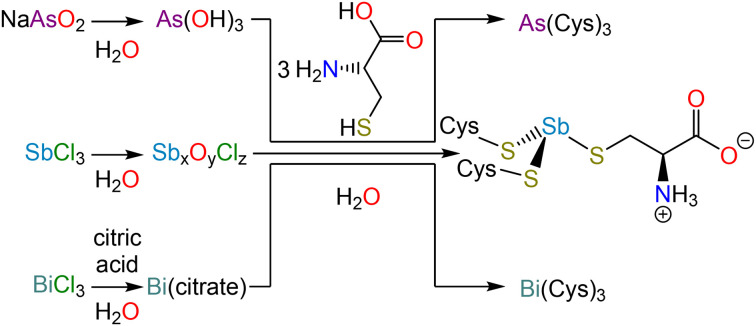
Syntheses of Pn(Cys)_3_ (Pn = As, Sb, Bi).

In the interpretation of these macromolecular structures, allusion is almost invariably made to high-resolution crystal structures of small molecule analogs. Surprisingly, the structures of the most relevant analogs, namely As(Cys)_3_, Sb(Cys)_3_, and Bi(Cys)_3_, have not been satisfactorily solved. In this paper, we revisit the synthesis and isolation of these three species (Scheme 1), report the crystal structure of each, and conduct a comparative analysis across the series. Not only will these structures be valuable for understanding the interaction of these elements with proteins, but they will also provide insight into the design of chelators, which have long been of interest for therapeutic decorporation but are now increasingly important given that medicinally valuable radionuclides of each of these elements are currently being explored (*e.g.*, ^72^As, ^77^As, ^119^Sb, ^213^Bi).^31–33^

## Experimental

### General methods

Reagents and solvents were purchased from commercial vendors and used as received unless otherwise specified. NMR spectra were collected using a Bruker Avance III HD 500 spectrometer equipped with a multinuclear Smart Probe. To obtain As(Cys)_3_, Sb(Cys)_3_, and Bi(Cys)_3_ solutions at concentrations high enough for NMR spectroscopic measurements, 0.015 mM solutions of each pnictine were prepared in 0.078 mM Na_2_CO_3_ in D_2_O. Melting point data were collected with an electrothermal Mel-Temp apparatus and a partial-immersion thermometer; temperatures are uncorrected. Elemental analysis was performed at the UC Berkeley College of Chemistry Microanalytical Facility. UV-visible absorption spectra were measured on a Shimadzu UV-2401PC dual-beam spectrophotometer.

### Synthesis of tricysteinylarsine, As(Cys)_3_

NaAsO_2_ (130 mg, 1 mmol) was dissolved in deionized water (5 mL). Separately, l-cysteine (364 mg, 3 mmol) was dissolved in a solution of concentrated nitric acid (100 μL) in deionized water (5 mL). The NaAsO_2_ solution was added to the solution of l-cysteine and a colorless solid immediately precipitated; the pH of the supernatant was approximately 5. The reaction mixture was placed on ice for 30 min. The solid was collected by vacuum filtration and washed with cold water. Yield: 308 mg, 70%. M.p. 191 °C (dec). % Found: C 24.71, H 4.08, N 9.56. % Calc. for C_9_H_18_AsN_3_O_6_S_3_: C 24.83, H 4.17, N 9.65. ^1^H NMR (500 MHz, 0.078 mM Na_2_CO_3_ in D_2_O, *δ*) 3.51 (dd, *J* = 7.1 Hz, 4.5 Hz, 1H), 3.06 (dd, *J* = 13.5 Hz, 3.6 Hz, 1H), 2.95 (br s, 1H) ppm. ^13^C{^1^H} NMR (125 MHz, 0.078 mM Na_2_CO_3_ in D_2_O, *δ*) 178.27, 57.22 ppm. To grow crystals suitable for X-ray diffraction, the reaction was repeated but instead of collecting the colorless precipitate by filtration, the mixture was treated with aqueous sodium carbonate until it fully dissolved. Slow addition of glacial acetic acid induced the formation of diffraction-quality crystals.

### Synthesis of tricysteinylstibine monohydrate, Sb(Cys)_3_·H_2_O

SbCl_3_ (228 mg, 1.0 mmol) was suspended in deionized water (5 mL), resulting in the immediate formation of a white precipitate. This solid was collected by vacuum filtration, washed with deionized water (3 × 5 mL) and dried by passing air through the filter cake. This solid was then suspended in deionized water (5 mL). Separately, l-cysteine (364 mg, 3 mmol) was dissolved in a solution of concentrated nitric acid (100 μL) in deionized water (5 mL) and added to the Sb-containing suspension. The reaction mixture was stirred at room temperature for 1 h. The solid was collected by vacuum filtration, washed with cold water, and dried by passing air through the filter cake. Yield: 320 mg, 66%. M.p. 185 °C (dec). % Found: C 21.89, H 3.93, N, 8.44. % Calc. for C_9_H_20_SbN_3_O_7_S_3_: C 21.61, H 4.03, N 8.40. ^1^H NMR (500 MHz, 0.078 mM Na_2_CO_3_ in D_2_O, *δ*) 3.70 (br s, 1H), 3.13 (dd, *J* = 13.2 Hz, 4.1 Hz, 1H), 3.03 (dd, *J* = 13.3 Hz, 7.0 Hz 1H) ppm. ^13^C{^1^H} NMR (125 MHz, 0.078 mM Na_2_CO_3_ in D_2_O, *δ*) 178.35, 59.08, 32.38 ppm. To grow crystals for X-ray diffraction, ethanol was allowed to diffuse in the vapor phase into the filtrate from the reaction mixture.

### Synthesis of tricysteinylbismuthine monohydrate, Bi(Cys)_3_·H_2_O

Bi(NO_3_)_3_·5H_2_O (240 mg, 0.49 mmol) was dissolved in deionized water (20 mL) containing citric acid (300 mg, 1.56 mmol). The pH of the mixture was adjusted to approximately 7 with NH_3 (aq)_ and the reaction was heated to 60 °C for 2 h. Separately, l-cysteine (210 mg, 1.73 mmol) was dissolved in a solution of concentrated nitric acid (100 μL) in deionized water (20 mL). The solution of l-cysteine was added to the Bi-containing solution, and the reaction mixture immediately turned yellow in color. The pH was adjusted to approximately 7 and the reaction was stirred at room temperature overnight. The yellow solid that forms over the course of the reaction was collected by vacuum filtration, washed with cold water, and dried by passing air through the filter cake. Yield 101 mg, 18%. M.p. 182 °C (dec). % Found: C 18.31, H 3.29, N 6.95. % Calc. for C_9_H_20_BiN_3_O_7_S_3_: C 18.4, H 3.43, N 7.15. ^1^H NMR (500 MHz, 0.078 mM Na_2_CO_3_ in D_2_O, *δ*) 4.17 (dd, *J* = 6.5 Hz, 3.9 1H), 3.88 (dd, *J* = 13.3 Hz, 6.3 Hz, 1H), 3.72 (dd, *J* = 13.2 Hz, 3.8 Hz, 1H) ppm. ^13^C{^1^H} NMR (125 MHz, 0.078 mM Na_2_CO_3_ in D_2_O, *δ*) 179.26, 61.23, 31.80 ppm. To grow crystals for X-ray diffraction, ethanol was allowed to diffuse in the vapor phase into the filtrate from the reaction mixture.

### X-ray crystallography

Crystals of As(Cys)_3_, Sb(Cys)_3_, and Bi(Cys)_3_·H_2_O were grown as described above, selected under a microscope, loaded onto a polyimide loop using Paratone-N, and mounted onto a Rigaku XtaLAB Synergy-S single-crystal diffractometer. Each crystal was cooled to 100 K under a stream of nitrogen. Diffraction of Cu Kα radiation from a PhotonJet-S microfocus source was detected using a HyPix6000HE hybrid photon counting detector. Screening, indexing, data collection, and data processing were performed with CrysAlisPro. The structures were solved using SHELXT and refined using SHELXL following established strategies.^34–36^ All non-H atoms were refined anisotropically. C-bound H atoms were placed at calculated positions and refined with a riding model and coupled isotropic displacement parameters (1.2 × *U*_eq_ for aryl groups and 1.5 × *U*_eq_ for methyl groups). O- and N-bound H atoms were located in the difference Fourier synthesis; their positional parameters were either constrained using a riding model or refined semi-freely and their isotropic displacement parameters were set equal to 1.5 × *U*_eq_ of the atom to which they are attached. In the case of Bi(Cys)_3_·H_2_O, one cysteinyl group and one water molecule were disordered across two positions and were modelled using similarity and rigid-bond restraints on bond lengths.

## Results and discussion

### Prior spectroscopic studies of Pn(iii) complexation by glutathione

NMR spectroscopic studies of the interaction of sodium arsenite with varying amounts of GSH demonstrated that the tripeptide binds to As(iii) in a 3 : 1 fashion.^14,37^ The most significant changes in both the ^1^H and ^13^C NMR chemical shifts and *J*_HH_ coupling constants occurred at the β position of the Cys residue, suggesting that the binding is through the S atom of the Cys residue. Mass spectrometric measurements confirmed the assignment of the product as As(SG)_3_.^14^ More recent EXAFS data were best fit with a model comprising three S-atom scatterers at 2.254(7) Å.^17^ Colorimetric and calorimetric methods were used to precisely measure the thermodynamic parameters describing the interaction between As(iii) and GSH.^38^

NMR and ESI-MS measurements also confirmed that GSH forms an Sb(SG)_3_ complex, binding through the cysteinyl S atoms.^15^ Although the complex is thermodynamically stable (pSb = 22.1; pM = –log[M], where [M] is the equilibrium concentration of unchelated unhydrolyzed metal ion in a pH 7.4 solution with a total metal ion concentration of 1 μM and a total ligand concentration of 10 μM), it still undergoes rapid exchange with free GSH at physiological pH (*k* ≈ 9000 s^−1^).

Finally, similar NMR studies of the interaction of Bi(iii) with GSH indicated that it similarly forms a Bi(SG)_3_ complex^16^ and EXAFS studies further confirmed that the ligand is bound through the cysteinyl S atoms.^26^ Early mass spectrometric studies suggested that a lower-coordinate complex was formed, but the solution that was analyzed had only been prepared with the Bi(iii) precursor and GSH in a 1 : 1 ratio.^39^ Using equilibrium concentrations under different conditions,^16^ the pBi value was calculated to be 26.5.^15^ Despite this thermodynamic stability, as with Sb, the complex remains labile and at physiological conditions the exchange rate was approximately 1500 s^−1^.

In all of the aforementioned GSH studies, the Pn(SG)_3_ complexes were not isolated, but rather generated and studied *in situ*.

### Synthesis and isolation of Pn(Cys)_3_

As(Cys)_3_ was described as early as the 1920s during the investigation of the reaction of As(v)-based antiparasitic drugs with thiols.^40,41^ Under harsh conditions, the arsonic acids would not only reduce to As(iii), but would lose the organic substituent and form As(Cys)_3_. This conclusion was supported by elemental analysis and iodometric titration of the reaction product, and comparison of those results to the data obtained from experiments on independently synthesized As(Cys)_3_, which could be prepared in water from As_2_O_3_ and l-cysteine,^42,43^ or in ethanol from AsCl_3_ and l-cysteine hydrochloride.^44^ In reinvestigating this substance, we found NaAsO_2_ (sodium *meta*-arsenite) to be a convenient starting material given that it dissolves readily in water. All of these reactions most likely proceed through the same As(OH)_3_ intermediate.^42,43^ As in those previous reports, we observed that, when l-cysteine is added to the As(iii) solution, As(Cys)_3_ rapidly precipitates. This solid can be recrystallized by dissolving the precipitate in an aqueous Na_2_CO_3_ solution and then slowly reacidifying with glacial acetic acid.

As described above for As-containing species, the reduction of Sb(v)-based antiparasitic drugs by biological thiols motivated early studies of Sb(SR)_3_ compounds.^45^ In practice, we found the most effective starting material for Sb(Cys)_3_ to be freshly hydrolyzed SbCl_3_. Addition of SbCl_3_ to water results in the immediate precipitation of a colorless solid (presumably a mixed antimony(iii) oxide/chloride) that could be isolated and washed extensively with water. This solid could then be suspended in a dilute nitric acid solution of l-cysteine. The originally suspended material was gradually converted to a suspension of analytically pure Sb(Cys)_3_·H_2_O.

In contrast to the previous approaches, by which we were unable to obtain the analogous Bi-containing product, we accessed Bi(Cys)_3_*via* a soluble, intermediate complex of Bi(iii) and citrate. This approach has been previously described,^46^ and involves the use of Bi(NO_3_)_3_·5H_2_O and citric acid to form a complex *in situ*, which is then allowed to react with l-cysteine to afford the final product. Combination of the solutions of l-cysteine and the Bi(iii) citrate complex produces a yellow color given that a charge transfer band develops as the Bi–S bonds form (Fig. S7†). Over the course of the reaction, Bi(Cys)_3_·H_2_O precipitates as a yellow solid.

### NMR spectroscopic characterization of Pn(Cys)_3_

The previously investigated, *in situ*-generated Pn(SG)_3_ complexes have solubility across a much greater range of pH values than the Pn(Cys)_3_ complexes. Although the decreased solubility of the Pn(Cys)_3_ species facilitates their crystallographic characterization (*vide infra*), it complicates their spectroscopic investigation. To perform NMR spectroscopic measurements, each of the Pn(Cys)_3_ was dissolved to a concentration of 0.015 mM in a D_2_O solution of 0.078 mM Na_2_CO_3_. The studies with the Pn(SG)_3_ complexes highlight that, at alkaline pH, rapid ligand exchange of can occur and the complexes slowly decompose.^15,16^ Indeed, over the course of hours, we see new signals develop in the NMR spectra of all of the Pn(Cys)_3_ complexes, but within the span of 1 h, we do not observe any changes. All of the values quoted below were obtained within 1 h. In the ^1^H NMR spectra, there are three observable signals: H_α_ and the diastereotopically split H_β1_ and H_β2_. As the atomic number of the Pn center increases, a systematic downfield shift of all three NMR signals is observed (Table 1). This trend is non-linear and a significantly larger change in the H_β_ proton shifts is observed on transitioning from Sb(Cys)_3_ to Bi(Cys)_3_, than from As(Cys)_3_ to Sb(Cys)_3_. The general downfield shift is consistent with an increase in electron transfer from the l-cysteine thiolate to the softer Pn(iii) centers and a consequent deshielding of nuclei of the thiolate ligands.

**Table 1:** Chemical shifts (ppm) of l-cysteine and Pn(Cys)_3_ (Pn = As, Sb, Bi) in alkaline D_2_O^a^

	^1^H chemical shifts (*δ*, ppm)		^13^C chemical shifts (*δ*, ppm)		
	H_α_	H_β1_/H_β2_	C_α_	C_β_	C_C=O_
Cys	3.25	2.87/2.55	59.28	29.76	179.29
As(Cys)_3_	3.51	3.06/2.95	57.2	—b	178.2
Sb(Cys)_3_	3.70	3.13/3.03	59.1	32.4	178.4
Bi(Cys)_3_	4.17	3.88/3.72	61.2	31.8	179.2

a NMR measurements were performed on 0.015 mM solutions of the analytes in 0.078 mM Na_2_CO_3_ in D_2_O.

b Signal not observed because of intermediate-rate chemical exchange.

For all three species, there is a narrowing of the chemical shift difference between the diastereotopic H_β_ protons as compared to free l-cysteine. For the latter, the H_β_ protons are split by 0.32 ppm, whereas the corresponding signals in the Pn(Cys)_3_ species are separated by 0.10–0.16 ppm. Another notable feature of the spectra is that there is a consistent sharpening of the signals as the family is descended. Whereas Sb(Cys)_3_ and Bi(Cys)_3_ exhibit sharp, well-resolved signals, ^3^*J*_αβ_ coupling cannot be resolved in the H_β_ signals of As(Cys)_3_. Similarly broad signals were observed in prior studies where As(Cys)_3_ was prepared *in situ*.^47^ This observation is consistent with the relative rates of ligand exchange observed across the Pn(SG)_3_ species.^15,16^ The impact of this exchange is most notably evident in the ^13^C{^1^H} NMR spectra. Sb(Cys)_3_ and Bi(Cys)_3_ each exhibit three sharp, well-resolved signals for C_α_, C_β_, and C_C=O_. In contrast, As(Cys)_3_ shows only two broadened signals for C_α_ and C_C=O_.

### X-ray crystal structures of Pn(Cys)_3_ (Pn = As, Sb, Bi)

**Fig. 1:**
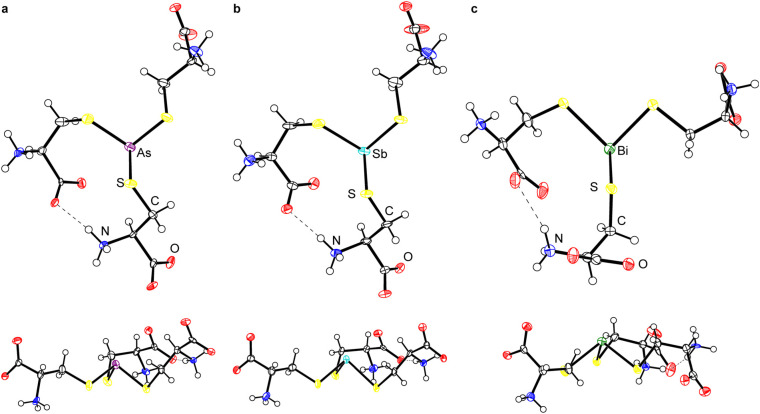
Thermal ellipsoid plots (50% probability level) of (a) As(Cys)_3_, (b) Sb(Cys)_3_, and (c) Bi(Cys)_3_. All structures are shown with the S_3_ plane parallel to (top) and perpendicular to (bottom) the plane of the page. For (c), the minor component of the disorder and the water of crystallization are omitted for clarity. Color code: As purple, Sb teal, Bi green, S yellow, O red, N blue, C black, H white spheres of arbitrary radius.

Although the crystallization of As(Cys)_3_ was described almost a century ago,^40,41^ its structure has never been determined. We obtained diffraction-quality crystals of As(Cys)_3_ by acidification of the base-homogenized reaction mixture with acetic acid. The compound crystallized in monoclinic Sohncke space group *I*2 (Table 2). The single molecule in the asymmetric unit resides on a general position and there are no crystallographically imposed relationships between bond metrics or on the overall symmetry of the molecule. The central As(iii) center is bound by three l-cysteine thiolates and all three ligands feature the amino acid in the zwitterionic ammonium carboxylate form (Fig. 1a). Although the H atoms in the final model were placed at calculated positions and refined using a riding model, residual electron density maxima for all three N-bound H atoms were located in the difference Fourier synthesis and the H-bonding pattern is consistent with the zwitterionic structure. Among these H-bonds is a strong intramolecular interaction between the carboxylate of one arm and the ammonium of another. The S–As–S angles range from 93.35(7)° to 97.89(7)°, and the As–S bond lengths range from 2.2392(17) Å to 2.2638(19) Å (Table 3).

**Table 2:** Crystallographic data collection and refinement parameters

	As(Cys)_3_	Sb(Cys)_3_	Bi(Cys)_3_·H_2_O
Formula	C_9_H_18_N_3_O_6_S_3_As	C_9_H_18_N_3_O_6_S_3_Sb	C_9_H_20_BiN_3_O_7_S_3_
FW	435.36	482.19	587.44
*T* (K)	104(6)	99.97(13)	99.99(10)
*λ* (Å)	1.54184	1.54184	1.54184
Crystal system	Monoclinic	Monoclinic	Orthorhombic
Space group	*I*2	*I*2	*P*2_1_2_1_2_1_
*a* (Å)	11.9981(3)	12.1577(8)	5.10700(10)
*b* (Å)	5.10005(13)	5.0648(4)	11.7830(3)
*c* (Å)	26.8857(7)	27.3420(15)	28.0118(7)
*β* (°)	93.718(2)	94.891(6)	
Volume (Å^3^)	1641.70(7)	1677.5(2)	1685.63(7)
*Z*	4	4	4
*ρ*_calc_ (Mg m^−3^)	1.761	1.909	2.315
Size (mm^3^)	0.14 × 0.04 × 0.03	0.17 × 0.05 × 0.02	0.1 × 0.05 × 0.01
2*θ* range (°)	6.59 to 136.466	6.49 to 136.204	6.31 to 140.15
Total data	9541	9272	6486
Unique data	2977	3062	2915
Parameters	203	203	298
Completeness (%)	99	100	100
*R*_int_ (%)	4.05	11.54	4.22
*R*_1_ (*I* > 2*σ*) (%)	3.91	5.53	3.12
*R*_1_ (all data) (%)	4.13	6.78	3.45
w*R*_2_ (*I* > 2*σ*) (%)	10.19	12.00	7.65
w*R*_2_ (all data) (%)	10.28	12.85	7.86
*S*	1.075	1.001	1.029
Flack *x*	0.00(4)	−0.016(19)	−0.043(18)

**Table 3:** Crystallographically refined bond metrics for Pn(Cys)_3_ (Pn = As, Sb, Bi)

Compound	Pn–S length	S–Pn–S angle	*χ*_1_ torsion angle^a^
As(Cys)_3_	2.2392(17)	93.35(7)°	−55.2(4)°
	2.2638(19)	97.89(7)°	63.5(4)°
	2.2405(17)	96.67(7)°	−49.1(4)°
Sb(Cys)_3_	2.427(4)	90.68(12)°	−51.4(10)°
	2.433(4)	96.39(12)°	68.4(10)°
	2.456(4)	90.74(13)°	−57.6(11)°
Bi(Cys)_3_^b^	2.542(2)	85.46(8)°	71.5(6)°
	2.544(3)	106.8(5)°/104.9(4)°	65.5(7)°
	2.49(3)/2.60(3)	88.9(4)°/94.3(4)°	76.0(16)°/66.0(14)°

a Measured as the N–C_α_–C_β_–S torsion.

b For disordered components, both values are provided, separated by a slash.

Because of this extensive network of H-bonds, however, we interpret the overall conformation of the molecule with caution. The *χ*_1_ angles for the three thiolate ligands, −55.2(4)°, 63.5(4)°, and −49.1(4)°, reflect the fact that two of them assume the typically most favorable *gauche*(+) conformation, but one assumes the typically least favored *gauche*(−) conformation. This unfavorable conformation is presumably assumed to maximize intra- and intermolecular H-bonding interactions. One of the most notable features of the structure is that the molecule is present in an “all-*endo*” conformation. This terminology indicates the relative dispositions of the As atom and the C_β_ atom of a single arm with respect to the plane defined by the three As-bound S atoms.^30^ In the *endo* conformation, the As and C_β_ atoms are on the same side of the plane; in the *exo* conformation, they are on opposite sides of the plane. These conformations have been discussed with relevance to peptide/protein binding,^30,48–50^ as well as small-molecule dipnictine compounds.^51–53^

**Fig. 2:**
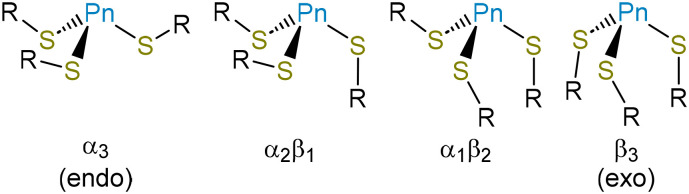
Schematic depiction of the different conformers of Pn(SR)_3_ compounds.

The *endo*/*exo* descriptors appear to have been originally introduced to describe three-fold (or approximately three-fold) symmetric molecules,^30^ and the terms provide a compellingly graphic description of whether the metal(loid) is buried within the three thiolates (*endo*) or projects out from them (*exo*). In the case of non-axially symmetric complexes, however, the metal(loid) can be on the same side of the S_3_ plane as some C_β_ atoms, and on the opposite side of the plane from others. This possibility has led some authors to reasonably ascribe an *endo*/*exo* designation to each ligand.^49,50^ In this paper, we allude to the α/β terminology used to describe atropisomers of porphyrins and use α to indicate a substituent where the S-bound C atom is on the same side of the S_3_ plane as the Pn atom and β to indicate a substituent where the S-bound C atom is on the opposite side of the S_3_ plane from the Pn atom (Fig. 2). The α_3_ conformation corresponds to *endo* and β_3_ conformation to *exo*. Importantly, mixed α_2_β_1_ and α_1_β_2_ conformations are possible. The structure of As(Cys)_3_ shows it to be an α_3_ conformer, *i.e.*, it assumes an *endo* conformation.

Sb(Cys)_3_ formed crystals that were isomorphous with those of As(Cys)_3_ (Fig. 1b). Although the overall features of Sb(Cys)_3_ in the solid state are therefore broadly analogous to those described above for As(Cys)_3_, there are variations in the specific bond lengths and angles. Most notable is the increase in Pn–S bond lengths (2.427(4), 2.433(3), and 2.456(4) Å), as expected with the larger-radius Sb atom. The three crystallographically distinct S–Sb–S angles within this molecule (90.68(12)°, 96.39(12)°, and 90.74(13)°) are generally smaller than those of the lighter congener, consistent with the lesser degree of hybridization in the Pn-based hybrid atomic orbitals used to form the Sb–S bonds. The largest bond angle of 96.39(12)° corresponds to the two cysteinyl ligands that are engaged in the strong intramolecular H-bond described above. As with As(Cys)_3_, Sb(Cys)_3_ crystallizes as the α_3_*endo* conformer.

Bi(Cys)_3_ crystallized from the aqueous reaction mixture in a form that is distinct from that of the lighter congeners (Fig. 1c). Most notably, it crystallized as a hydrate. The crystal structure belongs to the orthorhombic Sohncke space group *P*2_1_2_1_2_1_. A search of the CSD reveals that this structure has been reported previously,^46^ but it suffered from significant shortcomings in the refinement. We therefore proceeded with a redetermination and were able to successfully model one of the l-cysteine ligands as disordered across two rather disparate locations in an approximately 1 : 1 ratio. This unmodeled disorder appears to be one of the primary contributors to the poor refinement of the prior structure. In our final model, the average Bi–S bond length is 2.54(5) Å (the number in parentheses is the standard deviation of the averaged bond lengths). The S–Bi–S bond angles range from 85.46(8)° to 106.8(5)°. It too crystalizes in the α_3_*endo* conformation.

Access to this series of Pn(Cys)_3_ structures for the heavy pnictogens allows us to systematically compare them for the first time (Table 3). As expected on the basis of the increase in the size of the Pn atom, the Pn–S bond length systematically increases. The sum of the S–Pn–S angles steadily decreases towards 270° as the atomic number of the pnictogen increases. The heavier p-block elements have a decreased propensity for hybridization, and so the Pn-based atomic orbitals used to form the Pn–S bonds become increasingly enriched in p character and the individual S–Pn–S angles draw closer to 90°. The amino acid ligands do not uniformly assume the most sterically favorable *gauche*(+) conformation, with other conformations being taken in order to maximize either intramolecular or intermolecular H-bonding. Across the series, however, all compounds assume an α_3_*endo* conformation.

### Small-molecule structural comparison

**Fig. 3:**
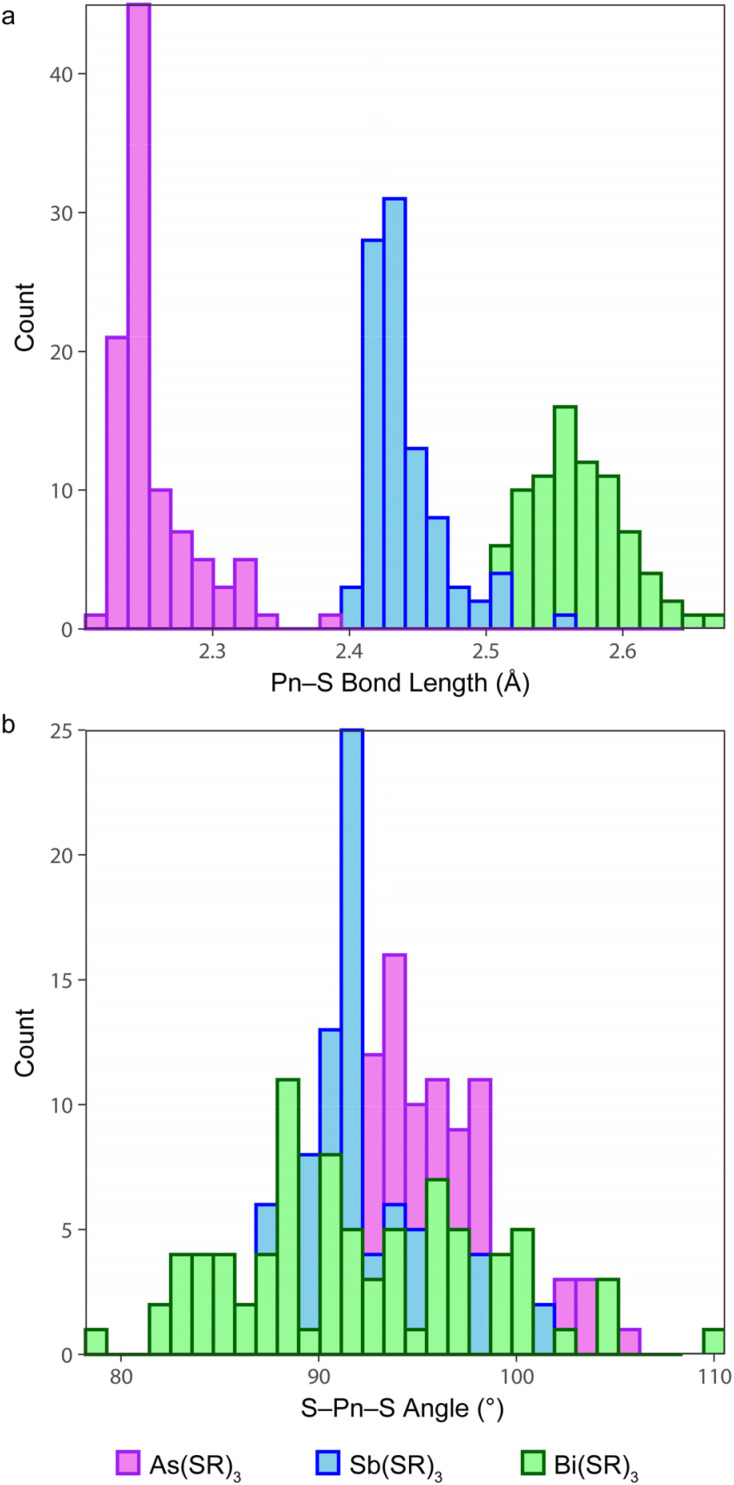
Distribution of (a) Pn–S bonds and (b) S–Pn–S bond angles for small-molecule As(iii), Sb(iii), and Bi(iii) trithiolate complexes deposited in the Cambridge Structural Database.

Early examples of Pn(iii) trithiolates heavily featured aryl and substituted aryl groups, such as As(SPh)_3_,^54^ Sb(STol)_3_,^55^ Sb(SAr′)_3_ (Ar′ = 2,4,6-^i^Pr_3_C_6_H_2_),^56^ Sb(SMe)(S_2_C_6_H_4_),^57^ Bi(SC_6_F_5_)_3_,^58^ and Bi(SAr′′)_3_ (Ar′′ = 2,4,6-^*t*^Bu_3_C_6_H_2_).^59^ The CSD was systematically searched for structures with a Pn(SC)_3_ fragment (*i.e.*, a substructure search) in which the Pn center makes exactly three connections, and each S atom makes exactly two connections. The returned CSD entries were then manually reviewed to restrict the final list to trithiolate complexes (Table S1†). For each of the Pn elements, the distributions of the Pn–S bond lengths and S–Pn–S angles are displayed in Fig. 3. In instances where *Z*′ > 1, all crystallographically inequivalent bond metrics were included in the analysis.

The Pn–S bond lengths of the Pn(Cys)_3_ species reported here fall well within the range of values reported for other small molecules. Strikingly, the vast majority of the compounds assume the α_3_*endo* conformation. Only in six instances did the compound assume a β_3_*exo* conformation. Each of the six instances falls into one of two categories. For three of the compounds (LAFBUS,^60^ DALBAX,^61^ and YUPZER^62^), the three arms of the ligand are covalently bound together and are too short to permit an *endo* conformation. In the other three cases, the organic substituents of the ligands fold back to allow extensive intermolecular π-stacking (QENTEJ),^63^ intermolecular chalcogen bonding (MTBTSB10),^57^ or intramolecular chalcogen bonding (OKOSAI).^64^

### Macromolecular structural comparison: peptide complexes

**Fig. 4:**
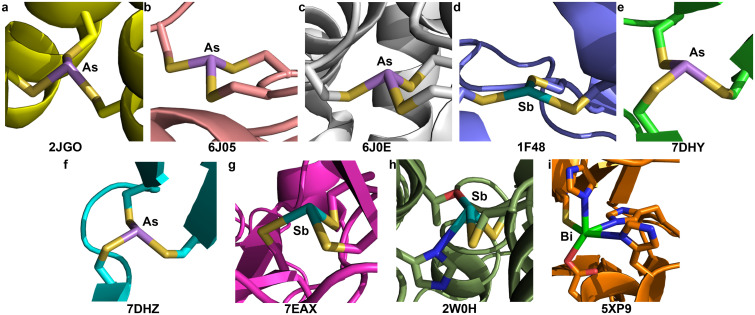
Macromolecular structures deposited in the Protein DataBank that feature Pn(Cys)_3_, or related, units. Underneath each is provided the PDB ID number. (a) As(iii) bound to three equivalents of the “Coil Ser L9C” (CSL9C) peptide. (b) As(iii) bound to ArsR from *Acidithiobacillus ferrooxidans* (AfArsR). (c) As(iii) bound to ArsR from *Corynebacterium glutamicum* (CgArsR). (d) Sb(iii) bound to ArsA from *Escherichia coli* (EcArsA). (e) As(iii) bound to the G245S mutant of p53. (f) As(iii) bound to the R249S mutant of p53. (g) Sb(iii) bound to the V272M mutant of p53. (h) Sb(iii) bound to trypanothione reductase from *Leishmania infantum*. The Sb center is only bound to two Cys residues; a Thr and a His are close enough to propose their interaction with the metalloid. (i) Bi(iii) bound to New Delhi metallo-β-lactamase 1 (NDM-1) from *Klebsiella pneumoniae*. The Bi center is only bound to one Cys residue; an Asp and multiple His are close enough to propose their interaction with the metalloid. In instances where there is more than one Pn(iii) center in the asymmetric unit, only one is shown.

Although many biophysical and biochemical studies have been performed to probe the interaction of As(iii), Sb(iii), or Bi(iii) with different proteins related to detoxification/biotransformation of these species, or their antiparasitic/anticancer mechanism of action, there have been relatively few macromolecular structures of heavier Pn(iii) centers bound to proteins. The Pecoraro group worked extensively on self-assembling coiled coil polypeptides that could be programmed with amino acid substitutions to reliably form structures that are preorganized for metal-ion binding. Using “Coil Ser L9C” (or CSL9C), they crystallized the As(CSL9C)_3_ complex in which one l-cysteine thiolate from each chain binds to the As(iii) center (PDB: 2JGO, Fig. 4a).^30^ The complex assumes an α_3_*endo* conformation with an average As–S bond length of 2.29(4) Å and an average S–As–S angle of 91(2)°. Here and below, the number in parentheses is the standard deviation of all the values included in calculating the mean. Importantly, even with the high-resolution (1.8 Å) data that were collected, restraints on the As–S distances were used during refinement. The restraint target of 2.25(5) Å was based on EXAFS data,^27^ and agree well with the values from our As(Cys)_3_ structure.

### Macromolecular structural comparison: As and Sb binding to Ars proteins

Decades of research have elucidated much of the biochemistry that underlies the As resistance conferred by genes encoded in *ars* operons. ArsR is a metalloregulatory protein that controls expression of these genes in response to As(iii) exposure. Regulation relies on As(iii)-induced dimerization but it was only in 2019 that crystal structures of As(iii)-bound ArsR proteins were reported (PBD: 6J05 and 6J0E, Fig. 4b and c).^65^ Proteins from two different species were crystallized: *Acidithiobacillus ferrooxidans* (AfArsR) and *Corynebacterium glutamicum* (CgArsR). In both structures, As(Cys)_3_ motifs are present. In the refinement of the As(iii)-bound CgArsR, two As(iii) centers are bound in the two different chains and the As–S bond lengths were restrained with a target value of 2.25 Å based on EXAFS studies reported earlier.^66,67^ The averaged refined As–S bond length and S–As–S angle across both As(iii) centers were 2.31(4) Å and 97(8)°, respectively. Both As centers assumed an α_3_*endo* conformation. For AfArsR, there was only one As(iii) center bound to the protein, and the final average values were 2.24(3) Å for As–S bond lengths and 98(7)° for S–As–S angles. The complex is in an α_3_*endo* conformation, but it is noteworthy that one of the C_β_ atoms is nearly in the S_3_ plane.

In the *ars* operon, ArsA functions as an ATPase that drives the pumping of As(iii) and Sb(iii) from the cytosol. From biochemical evidence, it was determined that As(iii) and Sb(iii) bind C113, C172 and C422.^19^ Crystallization of ArsA from *Escherichia coli* (EcArsA) was performed from a solution containing 2 mM NaSbO_2_.^68^ The crystals that formed contained one Sb center that was modeled as an H_2_SbO_3_^−^ anion (Sb–O lengths of 2.14, 2.11, and 1.90 Å), as well as three additional Sb atoms near C113, C172, and C422 (PDB: 1F48, Fig. 4d).^69^ Only one of these Sb centers coordinates to all three Cys residues; the others have additional Cl^−^ ions and water molecules bound as ligands. The average of the Sb–S distances of the Sb(Cys)_3_ unit is 2.62(6) Å. The complex has very low pyramidality, with S–Sb–S angles of 144°, 90°, and 104°. The long bond lengths and wide bond angles deviate strongly from the small molecule data described above.

### Macromolecular structural comparison: As and Sb binding to p53

At present, ATO is only approved for the clinical treatment of APL, but as a clinically approved agent, it is often included in screens for activity against other cancers. A recent study found that ATO was able to rescue p53 mutants with compromised structural integrity and crystallographic studies demonstrated that As(iii) can bind to a cryptic Cys_3_ allosteric site in the DNA-binding domain of two different mutants: G245S (PDB: 7DHY, Fig. 4e) and R249S (PDB: 7DHZ, Fig. 4f).^70^ In both cases, crystals of the mutated protein were soaked in an As(iii) solution. The As-bound G245S crystallized with four copies of the protein the asymmetric unit, and all four featured the As(Cys)_3_ unit. The As–S bond lengths were relatively consistent with an average value of 2.23(3) Å. This value agrees well with our As(Cys)_3_ structure and the aggregated small-molecule data described above. The average S–As–S angle was 109(8)°, although there was a large spread, with minimum and maximum values of 100.1° and 125.3°, respectively. The As-bound R249S crystallized with two copies of the protein in the asymmetric unit and both featured a similar As(Cys)_3_ unit. The average As–S bond length of 2.24(4) Å again agreed with our As(Cys)_3_ structure, and the average S–As–S angle of 110(8)° was again much greater. Across all As(Cys)_3_ units of both mutants, the α_1_β_2_ conformer was observed.

Appreciating the similarities that could be present between the previous experiments and the reaction of Sb(iii) with p53 mutants, a crystal structure of the V272M mutant was solved after soaking in a solution of potassium antimony tartrate, a source of Sb(iii) (PDB: 7EAX, Fig. 4g).^71^ Four copies of the protein are present in the asymmetric unit and an Sb atom was fit into the positive density of each. The Sb is located in the same Cys_3_ binding pocket described above for the As-bound mutants. The final averaged Sb–S bond lengths and S–Sb–S angles are 2.97(1) Å and 98(3)°, respectively. These values differ significantly from those of Sb(Cys)_3_ and the other Sb(SR)_3_ small molecules. It is possible that the Sb–S bond lengths in the protein structure are indeed elongated, but the discrepancy is sufficiently great that it merits either further investigation or discussion. It is also noteworthy that all four copies of the protein feature the α_1_β_2_ conformer, but the one α ligand features a highly acute Sb–S–C angle of 70°.

### Macromolecular structural comparison: Sb binding to trypanothione reductase

The interaction of Sb(iii) with trypanothione and trypanothione reductase has long been implicated in the mechanism of antileishmanial action of the pentavalent antimonials.^20^ Crystals of trypanothione reductase from *L. infantum* were soaked in a solution that contained potassium antimony tartrate, a source of Sb(iii). This structure (PDB: 2W0H, Fig. 4h) will not be discussed in detail because it does not have a Cys_3_ binding site, but it is noteworthy that the final model does have two Cys residues with Sb–S distances of 2.77 Å and 2.95 Å and an S–Sb–S angle of 96.1°. As in the ArsA structure above, the Sb–S bond lengths are much longer than those in the small-molecule structures collected here.

### Macromolecular structural comparison: Bi binding to Cys_3_ motifs

A complex of the peptide TRI L16C with Bi(iii) was never crystallized, but was investigated using XAS.^29^ The EXAFS of Bi(TRI L16C)_3_ was best fit with a model including three S atoms at a distance of 2.54 Å, a value in excellent agreement with our Bi(Cys)_3_ complex. There are very few protein structures in the PDB that feature Bi and none have the metal ion bound to a Cys_3_ unit. Crystals of New Delhi metallo-β-lactamase 1 (NDM-1) from *Klebsiella pneumoniae* were leached of the Zn(ii) that they contained and then soaked in a Bi(iii) solution.^72^ The resulting crystals (PDB: 5XP9, Fig. 4i) featured a Bi(iii) center interacting with a variety of coordinating residues, including His, Asp, and Cys. The Bi–S distance was 2.70 Å (averaged across the two disordered positions of the Bi). This distance is longer than those in the small-molecule structures described here, but the nature of the coordination sphere is also quite different.

## Conclusion

Despite the ability of l-cysteine to form well-defined complexes of As(iii), Sb(iii), and Bi(iii), and the importance of the Cys_3_ binding motif in the biological chemistry of these elements, the structures of the Pn(Cys)_3_ species had gone undetermined (Pn = As and Sb), or were in need of revision (Pn = Bi). In the absence of this information, structural biology studies had relied on small-molecule Pn(SR)_3_ models featuring R groups that often deviated significantly from those of the biological ligands. We extended our structural analysis beyond trends across the family and demonstrated that the bond metrics of this triad of compounds agree well with those of Pn(iii) trithiolate complexes deposited in the CSD. We also retrieved from the PDB all those structures that feature Pn(Cys)_3_ (Pn = As, Sb, and Bi) units. In a number of the original papers describing these protein structures, the authors indicate that they restrained the metal(loid) coordination environment based on the bond metrics of less biologically relevant Pn(SR)_3_ compounds. We anticipate that the presently described crystallographic data will be of use to structural biologists seeking more accurate models upon which to base their restraints. Analysis of the panel of refined protein structures revealed that in many cases there were significant deviations from the Pn–S bond lengths, S–Pn–S angles, and overall molecular conformation of the small-molecule structures. In some cases, the discrepancy may indicate a shortcoming of the macromolecular model. More importantly, however, it may be the case that some of the discrepancies highlight specific features of the protein–metal(loid) interaction where evolution specifically selected for a distorted coordination sphere. The Pn(Cys)_3_ structures reported here allow us to cleanly establish the baseline from which those distortions occur. We and others will also be using these structures in the design of chelators for heavy group 15 elements.

## Author contributions

S. E. H. performed experiments. S. E. H. and T. C. J. analyzed the data and wrote the manuscript.

## Data availability

Crystallographic data for As(Cys)_3_, Sb(Cys)_3_, and Bi(Cys)_3_·H_2_O have been deposited at the Cambridge Crystallographic Data Centre, under deposition numbers CCDC 2380696–2380698, and can be obtained from https://www.ccdc.cam.ac.uk/structures/. NMR spectra and Refcodes for CSD structures used for statistical analysis are available in the ESI† as a PDF document.

## Conflicts of interest

There are no conflicts to declare.

## Supplementary Material

DT-053-D4DT02476A-s001

DT-053-D4DT02476A-s002
