# Supplementary material for: Synthesis and structural characterization of the heavy tricysteinylpnictines, models of protein-bound As(iii), Sb(iii), and Bi(iii)
Source: Dalton Trans. 2024 Nov 26;53(47):18890–901. doi: 10.1039/d4dt02476a (PMC11590777; doi:10.1039/d4dt02476a)
Supplement: DT-053-D4DT02476A-s001 [file DT-053-D4DT02476A-s001.pdf]

Supplementary Information (SI) for Dalton Transactions.
This journal is © The Royal Society of Chemistry 2024

Electronic Supplementary Information for

Synthesis and structural characterization of the heavy tricysteinylpnictines, models of protein-bound As(III), Sb(III), and Bi(III)

Sophia E. Hollow[a] *and Timothy C. Johnstone**[a]

[a] Department of Chemistry and Biochemistry, University of California Santa Cruz, Santa Cruz, California 95064, United States.

Correspondence: johnstone@ucsc.edu

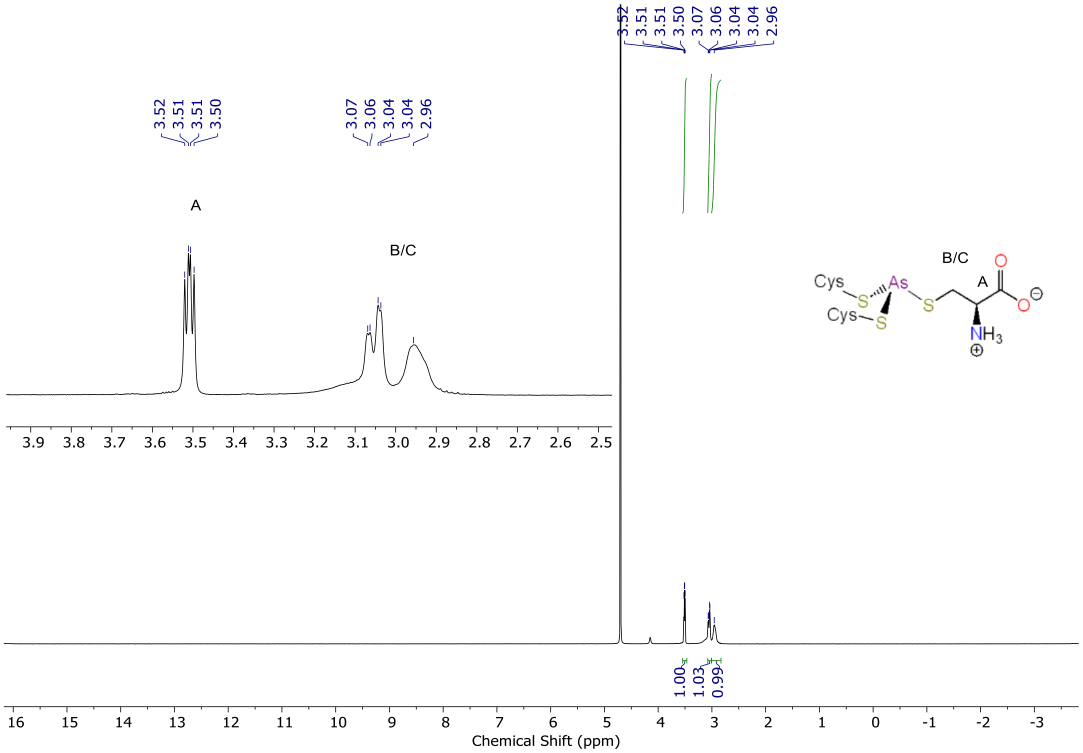

Figure S1. ^1H NMR spectrum (D_2O, 500 MHz, 0.078 mM Na_2CO_3) of As(Cys)$_3$ (0.015 mM).

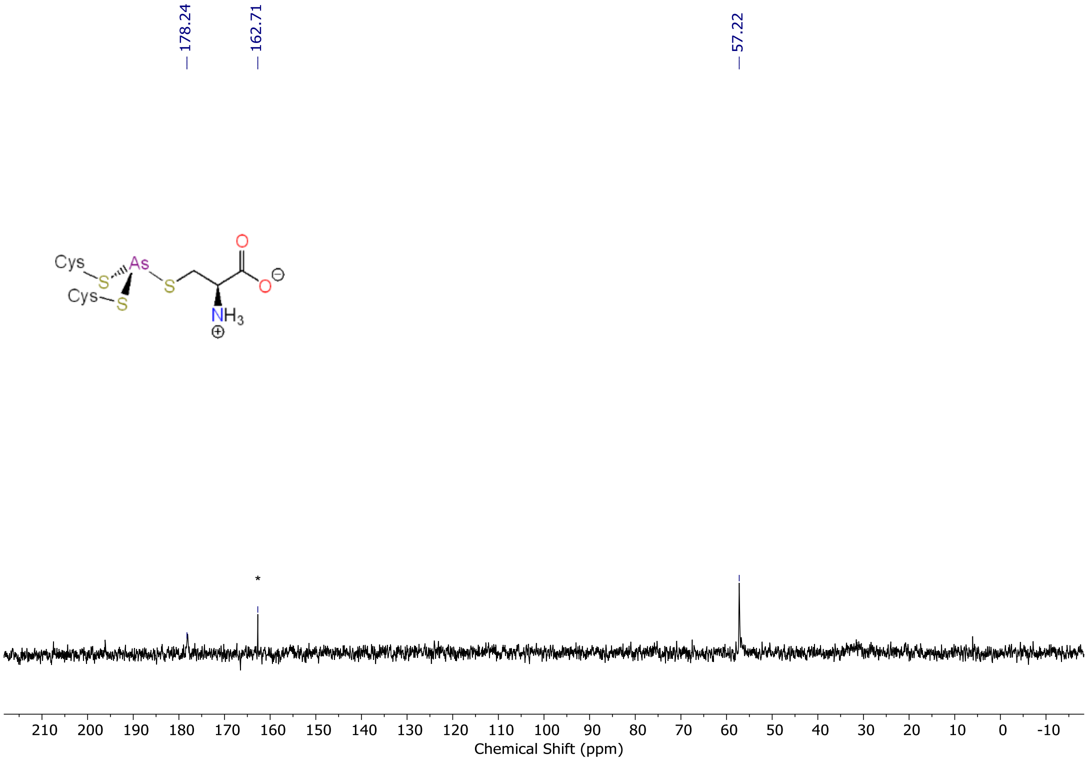

Figure S2. $^{13}C\{^1H\}$ NMR spectrum (D_2O, 125 MHz, 0.078 mM Na_2CO_3) of As(Cys)$_3$ (0.015 mM). The asterisk marks the carbonate/bicarbonate signal. The C_β signal is broadened by exchange to the point that it is not observed.

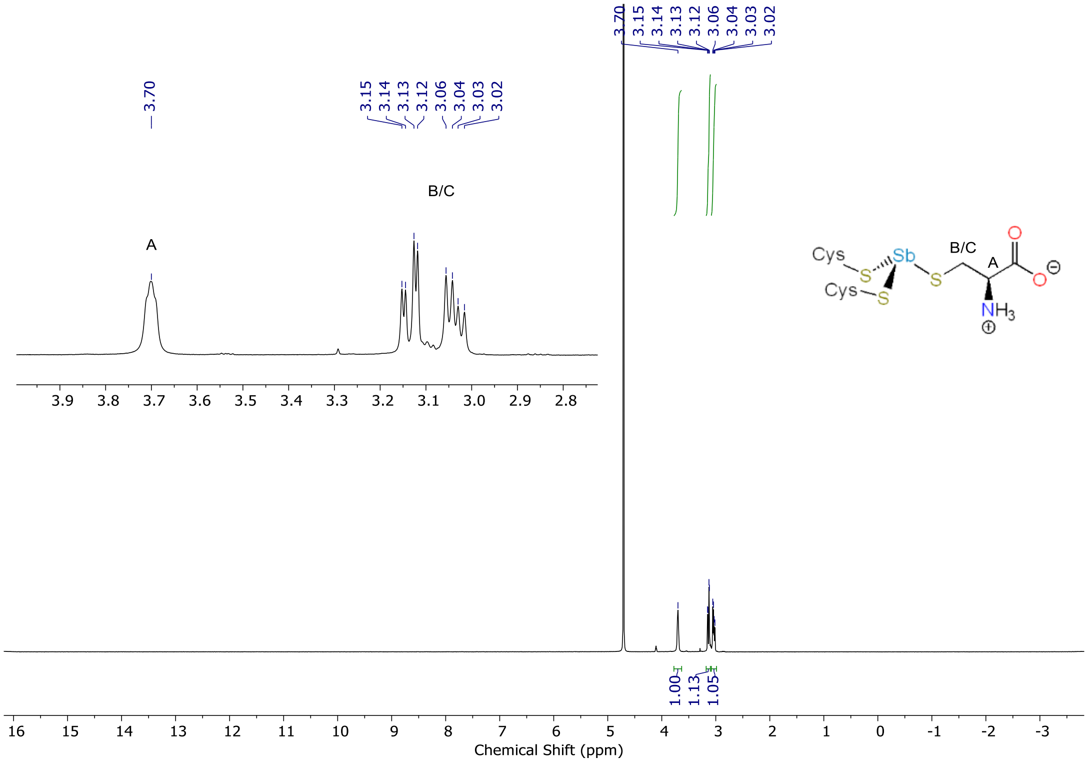

Figure S3. ^{1}H NMR spectrum (D_2O, 500 MHz, 0.078 mM Na_2CO_3) of $Sb(Cys)_3$ (0.015 mM).

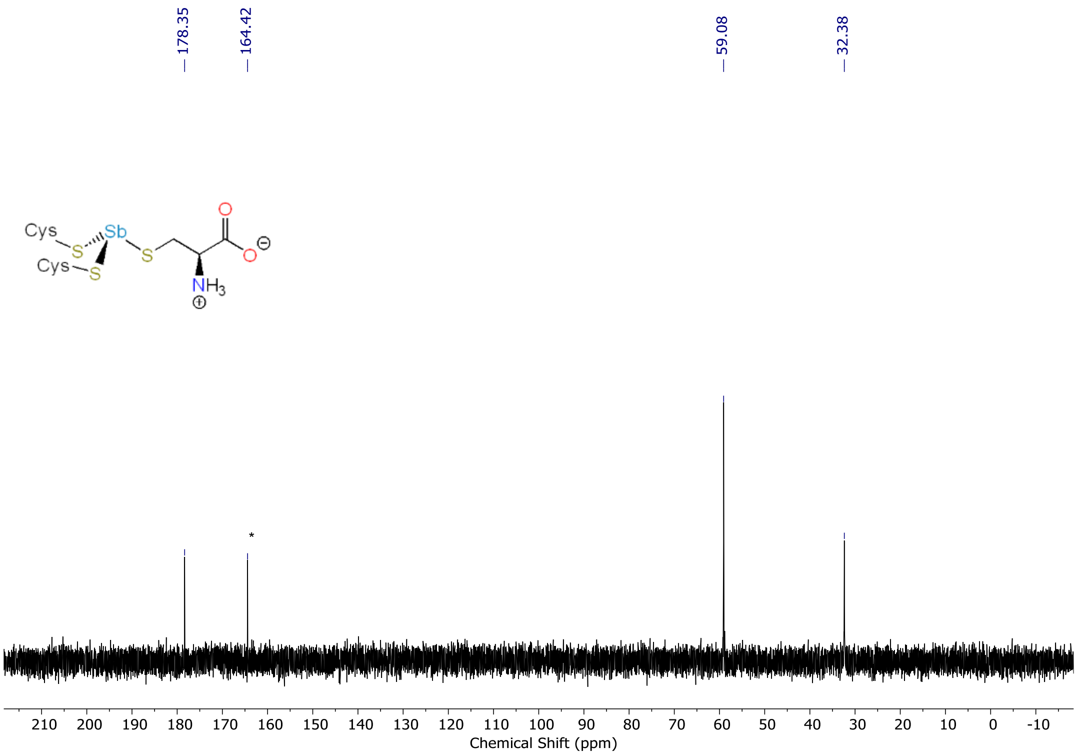

Figure S4. $^{13}C\{^{1}H\}$ NMR spectrum (D_2O, 125 MHz, 0.078 mM Na_2CO_3) of $Sb(Cys)_3$. (0.015 mM) The asterisk marks the carbonate/bicarbonate signal.

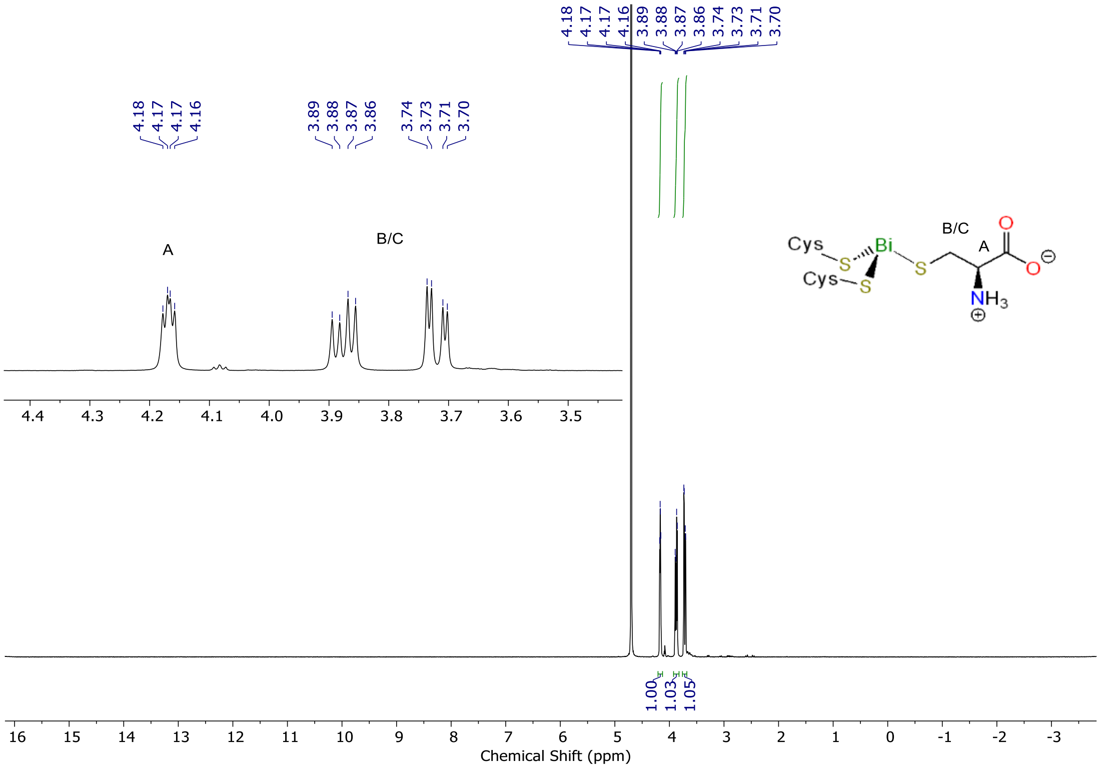

Figure S5. ^{1}H NMR spectrum (D_2O, 500 MHz, 0.078 mM Na_2CO_3) of $Bi(Cys)_3$ (0.015 mM).

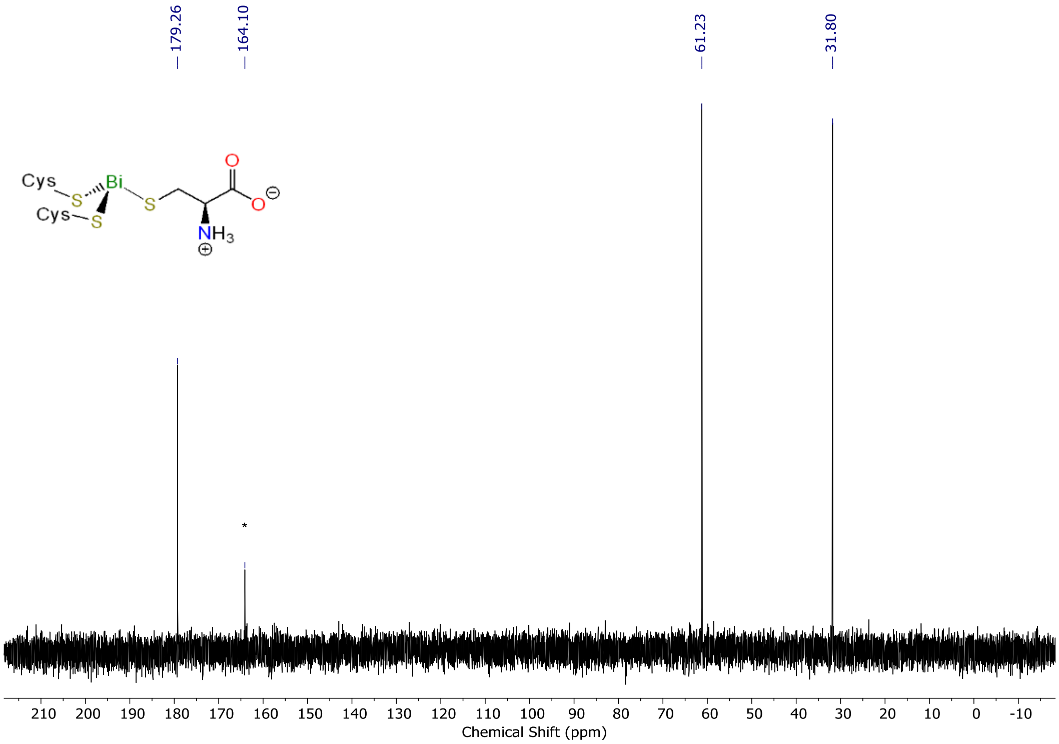

Figure S6. $^{13}C\{^{1}H\}$ NMR spectrum (D_2O, 125 MHz, 0.078 mM Na_2CO_3) of $Bi(Cys)_3$ (0.015 mM). The asterisk marks the carbonate/bicarbonate signal.

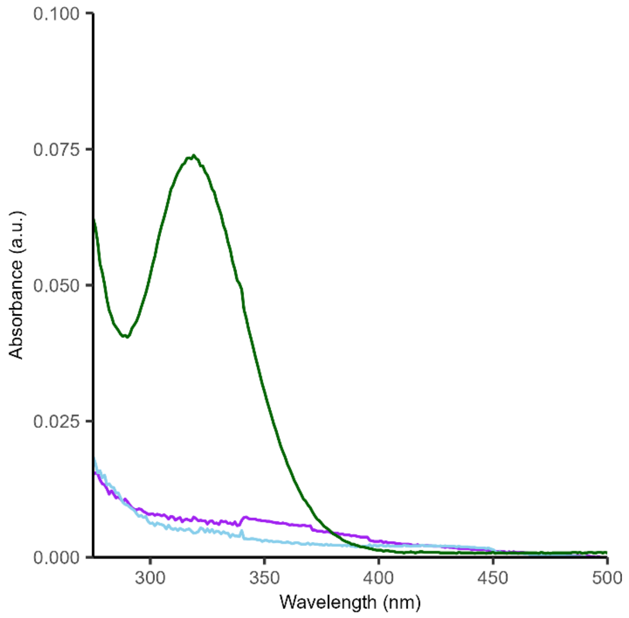

Figure S7. UV-Vis spectra for $Bi(Cys)_3$ (green), $Sb(Cys)_3$ (blue), $As(Cys)_3$ (purple). The concentration of the analyte was 30 nM and the sodium carbonate concentration was 156 nM.

Table S1. Refcodes for $Pn(SR)_3$ structures retrieved from the CSD.

**As**	**Sb**	**Bi**
BALFAX	BALGAY	CIYPIK
BALFEB	CIDWIV	IBEFIF
BALFIF	CUYKIQ	IMIVEF
BALFOL	DALBAX	MIWDUS
BALFUR	FATRIB	MIWFEE
CANWOG	MTBTSB10	OKIJUN
CATJOW	RALJUM	OKOSAI
CEDYOA	RALKAT	QEFVOO
CUBVIE	SIHKIE	RIPRUE
DEDLUT	SIPZIB	WATJUW
ETUXUP	SOPTOH	WOXFOG
FAQZUT	SQNLSB10	XUNHOE
FUWXAX	TEPREL	YUKRUR
GEMCEI	TIDGOC	ZEKZEY
HAMDIL	TIDHAP	ZUMSAB
IBAYAM	XUNJAS	ZUMSEF
KEKQOI	YUPZER	
LAFBUS	YUSFUN	
NEJQEZ	ZAZYII	
NEJRAW	ZAZYOO	
OGAYEB		
QENTEJ		
TUBCIG		
XUNHIY		
